# Mannose Receptor Mediates the Activation of Chitooligosaccharides on Blunt Snout Bream (*Megalobrama amblycephala*) Macrophages

**DOI:** 10.3389/fimmu.2021.686846

**Published:** 2021-08-02

**Authors:** Aotian Ouyang, Huabing Wang, Jianguo Su, Xiaoling Liu

**Affiliations:** ^1^Department of Aquatic Animal Medicine, College of Fisheries, Huazhong Agricultural University, Wuhan, China; ^2^Hubei Provincial Engineering Laboratory for Pond Aquaculture, Hubei Engineering Technology Research Center for Aquatic Animal Disease Control and Prevention, Wuhan, China; ^3^Engineering Research Center of Green Development for Conventional Aquatic Biological Industry in the Yangtze River Economic Belt, Ministry of Education, Wuhan, China

**Keywords:** chitooligosaccharide, mannose receptor, toll-like receptor, macrophages, *Megalobrama amblycephala*

## Abstract

Chitooligosaccharide (COS) is an important immune enhancer and has been proven to have a variety of biological activities. Our previous research has established an M1 polarization mode by COS in blunt snout bream (*Megalobrama amblycephala*) macrophages, but the mechanism of COS activation of blunt snout bream macrophages remains unclear. In this study, we further explored the internalization mechanism and signal transduction pathway of chitooligosaccharide hexamer (COS6) in blunt snout bream macrophages. The results showed that mannose receptor C-type lectin-like domain 4-8 of *M. amblycephala* (MaMR CTLD4-8) could recognize and bind to COS6 and mediate COS6 into macrophages by both clathrin-dependent and caveolin-dependent pathways. In the inflammatory response of macrophages activated by COS6, the gene expression of tumor necrosis factor (TNF)-α, interleukin (IL)-1β, and nitric oxide synthase 2 (NOS2) was significantly inhibited after MaMR CTLD4-8-specific antibody blockade. However, even if it was blocked, the expression of these inflammation-related genes was still relatively upregulated, which suggested that there are other receptors involved in immune regulation. Further studies indicated that MaMR CTLD4-8 and Toll-like receptor 4 (TLR4) cooperated to regulate the pro-inflammatory response of macrophages caused by COS6. Taken together, these results revealed that mannose receptor (MR) CTLD4-8 is indispensable in the process of recognition, binding, internalization, and immunoregulation of COS in macrophages of blunt snout bream.

## Highlights

1. MaMR CTLD4-8 can recognize and bind to COS.
2. MaMR CTLD4-8 mediates COS internalization into macrophages.
3. COS is internalized by clathrin- and caveolin-dependent pathways.
4. TLR4 and MaMR CTLD4-8 coordinately regulated the pro-inflammatory response.

## Introduction

Chitooligosaccharide (COS) is used as an important immune enhancer. It is a linear oligomer of N-acetylglucosamine or glucosamine linked by β-1,4 glycosidic bonds (1, 2). Essentially, COS is the degraded products of chitin/chitosan by acid hydrolysis, enzymatic degradation, or both. Its degree of deacetylation (DD) is >90%, degree of polymerization (DP) is <20, and molecular weight (MW) is <3,900 Da (3, 4). It is easily soluble in water and has the advantages of non-toxicity, non-sensitization, and excellent biocompatibility. In recent years, studies have shown that COS has a variety of biological effects, which can enhance immune effects and antimicrobial and antitumor activities (5–7).

As one of the most important components of innate immunity, macrophages play a key role in inflammation and host defense. The resting macrophages can be induced to activate M1-type macrophages under the stimulation of interferon (IFN)-γ and lipopolysaccharide (LPS), secreting a large number of pro-inflammatory factors (8), including interleukin (IL)-1, IL-6, and tumor necrosis factor (TNF)-α, and upregulate the messenger RNA (mRNA) level of inducible nitric oxide synthase (iNOS) [also called nitric oxide synthase 2 (NOS2)]. It has been reported that COS can activate the immune activity of macrophages; the recognition of COS by macrophages and the regulation of their functions are some of the important ways to exert immunomodulatory effects (9, 10). The immune regulation function of COS mainly reflects its activation and inhibition of different signal pathways, thereby regulating the production of NO and cytokines or mediators and finally acting on the innate immune system and the adaptive immune system (11–13). In mammals, COS has a specific signal pathway that regulates the activation of macrophages. It has been previously reported that the use of low-molecular weight chitohexaose (chitooligosaccharide hexamer, COS6) or COS with a degree of polymerization of only 1-6 can activate the nuclear factor (NF)-κB pathway of mouse macrophages and induce the production of nitric oxide (NO) and TNF-α (14, 15). There are also reports showing that COS or sulfurized COS modulates immune effects in mouse macrophages by regulating the mitogen-activated protein kinase (MAPK) and phosphoinositide 3-kinase (PI3K)/Akt signaling pathways (16, 17).

The mannose receptor (MR) is a type I transmembrane protein, which is an important member of the C-type lectin family and can realize rapid endocytosis through the cell membrane. From N-terminal to C-terminal of MR, there are cysteine-rich domain (CR), fibronectin type II domain (FNII), eight tandemly arranged C-type lectin-like domains (CTLDs), transmembrane domain, and short cytoplasmic tail. As the main carbohydrate recognition domain of MR, the CTLDs can mediate MR recognition of carbohydrate or carbohydrate complexes with D-mannose, L-fucose, and N-acetylglucosamine as terminal (18, 19). Combined with ligands, MR can initiate intracellular signaling cascades that promote or inhibit the production of NO and the expression of cytokines. The intracytoplasmic domain of MR is very short and does not contain signal transduction motif. Therefore, the involvement of MR in signal transduction requires the participation of other receptors for the expression of target genes.

Although there have been reports on the mechanism of COS to activate macrophages in mammals, there are still controversies. Previous studies have shown that the immunostimulatory effect of COS on macrophages depends on Toll-like receptor 4 (TLR4) (11), MR (20), CD14, or complement receptor 3 (CR3) (15). COS is an important immune enhancer in the field of aquaculture. However, the mechanism by which COS activates macrophages in aquatic animals is still unclear. In previous studies, we established a COS-activated blunt snout bream macrophage M1 polarization model and successfully expressed mannose receptor C-type lectin-like domain 4-8 of *M. amblycephala* recombinant protein (rMaMR CTLD4-8); the anti-MaMR CTLD4-8 polyclonal antibody has also been prepared. In order to further prove the mechanism of COS in activating blunt snout bream macrophages, in this study, we selected the kind of COS (COS6, molecular weight about 1,300 Da) that has the best inflammation activation effect as the experimental material. The results showed that the internalization of COS6 in the macrophages of blunt snout bream was mediated by MR CTLD4-8, and it was found that the immune stimulation of COS6 on macrophages was coordinated by MR CTLD4-8 and TLR4.

## Materials and Methods

### Fish Sampling

Blunt snout bream (*M. amblycephala*), ranging from 400 to 500 g in weight, were obtained from a fish farm located in Hubei Province, China, and kept in a recirculating freshwater system at 25°C–26°C with a natural photoperiod. The animals were fed twice per day with a commercial pellet diet (Haida, Hubei, China) amounting to 3% of body weight. The study was approved by the Institutional Animal Care and Use Ethics Committee of Huazhong Agricultural University (ID Number: HZAUFI-2021-0007).

### Isolation of Head Kidney Macrophages

Blunt snout bream head kidney macrophages were isolated as described previously with slight modifications (21). Briefly, fish were anesthetized with MS222 (Syndel Laboratories, Ltd., Canada), and the head kidney was removed aseptically and passed through a 100-μm mesh (Falcon, Becton Dickinson) in Leibovitz medium (L-15) (Invitrogen, USA) containing 2% fetal bovine serum (FBS) (Gibco, USA) and 200 IU/ml penicillin plus streptomycin (Amresco, USA). The resulting cell suspension was layered onto a 34%/51% Percoll (Pharmacia, Uppsala, Sweden) density gradient and centrifuged at 400 g for 30 min at 4°C. The interface was collected, and the cells were washed twice with L-15 at 400 g for 10 min at 4°C before being resuspended to 1 × 10^7^ cells/ml in L-15 containing 10% FBS.

### Indirect Immunofluorescence Assay

Indirect immunofluorescence was used to observe the binding of COS6 to MR CTLD4-8 in the head kidney macrophages from blunt snout bream. The macrophage (1 × 10^7^cells/ml in L-15) suspension is evenly cultured on the cell slide in 24-well plates. Cells were incubated with 0.05 mM fluorescein isothiocyanate (FITC)-labeled COS6 (Xianqiyue, China) at 28°C for 30 min. Cells were fixed with 4% paraformaldehyde for 10 min. Blocking solution 5% normal goat serum (Guge Biology, China) was added to minimize nonspecific fluorescence, and then anti-MaMR-CTLD4-8 immunoglobulin G (IgG) (9.6 μg/ml in blocking solution) was incubated at 28°C for 2 h, followed by incubation with Cy3-conjugated goat anti-mouse IgG (Abclonal, China) for 40 min. Nuclei were stained with 4′,6-diamidino-2-phenylindole (DAPI; Sigma, Germany) for 10 min. The membrane was stained by 1,1-dioctadecyl-3,3,3,3-tetramethylindodicarbocyanine (DiD; Beyotime, China) for 15 min. Anti-fluorescence quencher mounted and fixed cell slides. The slide is imaged by a confocal microscope (Leica TCS SP8, Germany).

### Direct Binding Assay of MaMR CTLD4-8 to Carbohydrates

The binding activity of MaMR CTLD4-8 to COS6 was performed by enzyme-linked immunosorbent assay (ELISA) according to the previous method with a little modification (22). COS6 and D-mannose (Man) at 80 μg/ml (100 μl) were used to coat a 96-well microtiter plate (Corning, USA). The plates were air-dried at 37°C and then blocked with 100 μl/well of 5% bovine serum albumin (BSA) in phosphate buffered saline with Tween 20 (PBST) for 2 h at 37°C. Wells were washed three times with PBST. Then, 100 μl rMaMR CTLD4-8 of different concentrations were added to the wells. After incubation at 37°C for 2 h, the plate was washed three times with PBST. After that, 100 μl mouse anti-glutathione-S-transferase (GST)-tag IgG (Abclonal, China) diluted 1:2,000 in 5% BSA were added to each well at 37°C for 1 h. Then, the plate was rewashed and incubated with 100 µl of goat anti-mouse IgG–horseradish peroxidase (HRP) conjugate (Biosharp, China) diluted 1:5,000 in 5% BSA at 37°C for 1 h. After washing with PBST three times, 100 µl of 3,3´,5,5´-tetramethylbenzidine (TMB) solution (Solarbio, China) was added to each well and incubated at 37°C for 10 min in the dark. The reaction was stopped by adding 100 µl of 2 M sulfuric acid per well. The absorbance was measured with an automatic ELISA reader (Tecan, Switzerland) at 450 nm. Mannose (Man) as a positive control; GST tag protein was employed as a negative control. The assay was repeated three times.

### Distribution of FITC-COS6 in Macrophages

The macrophage (1 × 10^7^cells/ml in L-15) suspension is evenly cultured on the cell slide in 24-well plates. Cells and 0.05 mM FITC-COS6 were incubated for 0, 2, 4, 6, 8, and 10 min at 4°C or 28°C. As above, the cells were fixed, DAPI and 1,1’-dioctadecyl-3,3,3’,3’-tetramethylindocarbocyanine perchlorate (DiI; Beyotime, China) stained, and observed by confocal microscope.

### Effect of Endocytosis Inhibitors on FITC-COS6 Internalization of Macrophages

The macrophage (1 × 10^7^cells/ml in L-15) suspension is evenly cultured on the cell slide in 24-well plates. The cells in each well were pretreated with chlorpromazine (CPZ, 40 μM), sucrose (300 mM), nystatin (100 μM), methyl-β-cyclodextrin (M-β-CD, 1 mM), 1,1’-dithiodi-2-naphthtol (IPA-3, 40 μM), and NSC23766 (hydrochloride, 40 μM) for 2 h at 28°C; according to the previous test method, it has been appropriately modified (23). The above inhibitors were purchased from MCE (China). Then, cells were incubated with 0.05 mM FITC-COS6 for 30 min. The final result was shown by the fluorescence images. After the cells were washed with PBS, lysed (Beyotime, China), add 100μ L supernatant to each well of 96-well plate, for fluorescence intensity was measured by fluorescence microplate at 488 nm. WCIF ImageJ software was used to analyze the co-localization of FITC-COS6 and DAPI-stained nucleus in macrophages.

### Effect of Anti-MaMR CTLD4-8 Antibody on FITC-COS6 Internalization of Macrophages

The macrophage (1 × 10^7^cells/ml in L-15) suspension is evenly cultured on the cell slide in six-well plates. Macrophages were preincubated with anti-MaMR CTLD4-8 IgG (4.8 μg/ml) for 2 h at 28°C. As a positive control, D-mannose (2 mg/ml) can effectively bind to MaMR CTLD4-8, and normal mouse IgG (4.8 μg/ml) is used as a negative control. Then, cells were incubated with 0.05 mM FITC-COS6 for 30 min at 28°C. The adherent macrophages were digested with trypsin containing 0.05% ethylenediaminetetraacetic acid (EDTA; Gibco, USA), and the digestion was terminated with serum after the cells were completely suspended. The cells were washed with PBS, centrifuged and resuspended to 1 × 10^6^ cells, and the fluorescence intensity at 488 nm was measured by flow cytometry (Berkam, USA). Or through the same processing method as above, the cells were incubated, washed, lysed, and mixed uniformly. Fluorescence intensity was measured by fluorescence microplate at 488 nm. The mean fluorescence intensity (MFI) of internalization from FITC-COS6 in macrophages was recorded. The percentage of FITC-COS6 intake under different pretreatments, which is the ratio of the MFI values measured from the blocked cells to those measured from the untreated cells, was plotted to reflect the efficiency of receptor endocytosis. Flow cytometry graphs shown in the *Results* section were representative data from at least three independent experiments.

### Western Blotting

Macrophages (1 × 10^7^ cells) were seeded into a six-well plate and cultured for 24 h. After 2 h preincubation with or without anti-MaMR CTLD4-8 IgG (4.8 μg/ml), COS6 (50 μg/ml) was added, giving a final volume of 1 ml. Incubated for 30 min, the cells were collected and lysed with a radioimmunoprecipitation assay (RIPA) lysis buffer (Beyotime, China) containing proteinase inhibitors on ice. The protein concentration was determined by a bicinchoninic acid (BCA) protein assay kit (Beyotime, China). Equal amounts of total protein were separated by 8%–12% sodium dodecyl sulfate–polyacrylamide gel electrophoresis (SDS-PAGE) gels and transferred onto nitrocellulose membranes (Millipore, Germany). The membranes were blocked in fresh 5% BSA dissolved in Tris-buffered saline with Tween 20 (TBST) buffer at room temperature for 1 h, then incubated with antibody TLR4 (1:500 dilution; HuaBio, China), TLR2 (1:500; HuaBio, China), and β-tubulin (1:4,000; Abclonal, China) overnight at 4°C. They were then washed three times with TBST buffer and incubated with HRP-conjugated goat anti-rabbit IgG (Abclonal, China) for 1 h at room temperature. Immunodetection was performed using enhanced chemiluminescence (ECL) reagents (GE Healthcare, USA).

### Quantitative Real-Time PCR Assay

Quantitative real-time PCR (qRT-PCR) was used to investigate the target gene expression patterns in different groups and different time points of macrophages after COS6 stimulation. Macrophages (1 × 10^7^ cells) were seeded into a six-well plate and cultured for 24 h.

To investigate the effect of MaMR CTLD4-8 after COS6 stimulated macrophages, cells were preincubated with anti-MaMR CTLD4-8 antibody and then cell samples were collected after stimulation with COS6 for 0, 3, 6, and 12 h. In order to investigate the effect of resatorvid (TAK 242; MCE, China) on COS6-stimulated macrophages, the cells were blocked with TAK242 and anti-MaMR CTLD4-8 antibody, and cell samples were collected after COS6 stimulation for 3 h. After the above method, the total RNA was extracted and cDNA was synthesized, and the following assay was prepared after uniform concentration.

Primers used for qRT-PCR of this experiment are given in **Table 1**. The qRT-PCR mixture reaction volume was 20 µl, containing 10 µl LightCycler^®^ 480 SYBR Green I Master, 7.4 µl ddH_2_O, 0.8 µl of each primer (10 mM), and 1 µl cDNA template. The reactions were performed using LightCycler^®^ 480 II (Roche Diagnostics GmbH, USA) according to the procedure as follows: preincubation at 95°C for 5 min, then 40 cycles at 95°C for 5 s, 55°C for 20 s, and 72°C for 20 s. Each sample was tested in triplicate. Specificity of the amplified target gene was assessed using dissociation curve analysis. The target gene relative expression levels *vs.* the β-actin gene (selected as the reference gene) were calculated according to the 2^-ΔΔCT^ method. To determine the relative fold change of the target gene at different time points, the expression value was normalized using the corresponding control group.

**Table 1:** Primers used in this study.

Primer name	Primer direction	Sequence (5’–3’)	GenBank	Application
MR	Forward	GATGGCAGTGGAGCAATGGA	KC495437.1	qRT-PCR
MR	Reverse	CTGGTGGAATGGTAGGAACAGA		
TNF-α	Forward	CCGCTGCTGTCTGCTTCA	HQ696609.1	qRT-PCR
TNF-α	Reverse	GCCTGGTCCTGGTTCACTCT		
IL-1β	Forward	GTGCCAGGTGCCAAGTAGC	KF245425.1	qRT-PCR
IL-1β	Reverse	AAGCCCAAGATATGCAGGAGT		
NOS2	Forward	ATTCAAGGGCAGCTTCCAGG	KJ668755.1	qRT-PCR
NOS2	Reverse	CAGGGGCAAAGTTTAAGGGC		
TLR4	Forward	CTGTCGTATGGTAGAGGTC	KR013051	qRT-PCR
TLR4	Reverse	TTCAGGTTTGAGTGGGTAA		
MyD88	Forward	GACAACAGGGATTAGACG	KP192128.1	qRT-PCR
MyD88	Reverse	TGGAACAGACTGAATACAAC		
β-Actin	Forward	ACCCACACCGTGCCCATCTA	ADV57164.1	qRT-PCR
β-Actin	Reverse	CGGACAATTTCTCTTTCGGCTG		

IL, interleukin; MR, mannose receptor; NOS, nitric oxide synthase; TLR, Toll-like receptor; TNF, tumor necrosis factor.

### Statistical Analysis

In the present study, statistical analysis and presentation graphics were carried out by the GraphPad Prism 8.0 software. Results were shown as mean ± SD from at least three independent experiments, and statistical significance was determined with ANOVA, followed by two-tailed Student’s t-test. The p–values <0.05 are considered statistically significant differences and p-values <0.01 as extreme difference.

## Results

### COS6**-Induced Pro-Inflammatory Response of Macrophages Involves MaMR CTLD4-8

**Figure 1:**
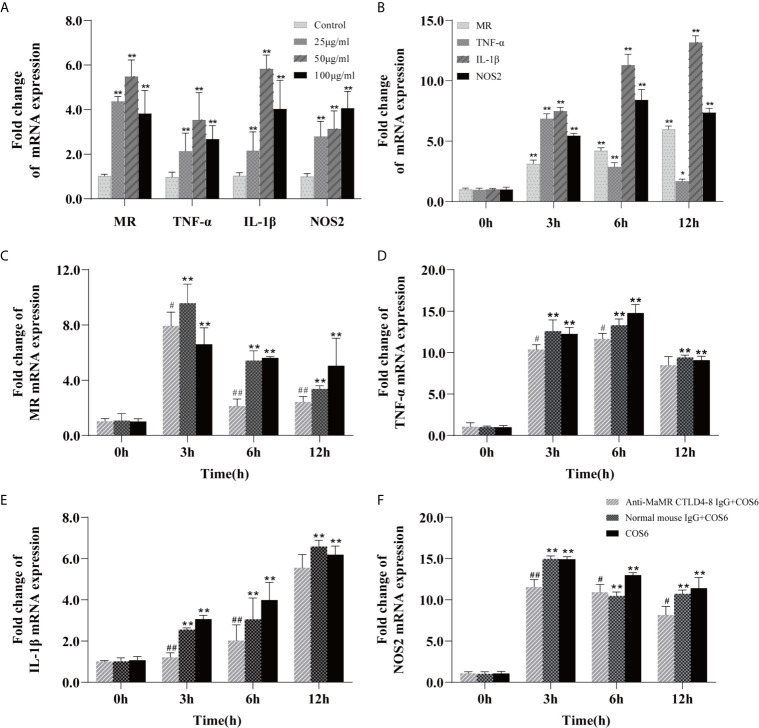
qRT-PCR analysis of the expression of mannose receptor (MR) and inflammation-related genes induced by chitooligosaccharide hexamer (COS6) in macrophages. **(A)** Gene expression in macrophages stimulated by COS6 for 6 h in 25, 50, and 100 μg/ml. **(B)** The macrophage gene expression was stimulated with 50 μg/ml COS6 at 0, 3, 6, and 12 h. Pre-blocking cells with or without anti-mannose receptor C-type lectin-like domain 4-8 of *Megalobrama amblycephala* (MaMR CTLD4-8) antibody, and the expression patterns of MR **(C)**, tumor necrosis factor (TNF)-α **(D)**, interleukin (IL)-1β **(E)**, and nitric oxide synthase (NOS)2 **(F)** were determined. The samples were analyzed at 0, 3, 6, and 12 h after 50 μg/ml COS6 stimulation. β-Tubulin was used as an internal reference. Each experiment was executed in triplicate. Data were shown as means ± SD (n = 3), with (*) p < 0.05 and (**) p < 0.01 *vs.* the 0 μg/ml or 0 h, (^#^) p < 0.05 and (^##^) p < 0.01 *vs.* the COS6 group (set as 1).

The M1 polarization model of COS-activated blunt snout bream macrophages has been successfully established in previous studies (24). On this basis, we first investigated the concentration gradient and time gradient of COS6-stimulated bream macrophages based on previous studies. After stimulation, these genes were significantly upregulated relative to the control group, and the expression levels of MR, TNF-α, and IL-1β were most significant at a COS6 concentration of 50 μg/ml (**Figure 1A**), and the expression levels of each gene were significantly upregulated at 3 h after stimulation (**Figure 1B**). After preblocking with anti-MaMR CTLD4-8 antibody, the gene expression after COS6 stimulation was quantitatively detected. Compared with the COS6 group, in the early stage of stimulation (12 h), MR (**Figure 1C**), TNF-α (**Figure 1D**), and IL-1β (**Figure 1E**) were most significantly blocked by antibody at 6 h, while NOS2 (**Figure 1F**) was most significantly blocked at 3 h. However, it is worth noting that the expression of inflammation-related genes and MR is still significantly upregulated compared to the 0 h control group. There may be other receptors or pathways involved in the regulation of COS6-induced macrophages. In conclusion, the blocking of MaMR CTLD4-8 antibody downregulated the expression of macrophage inflammation-related genes after COS6 stimulation, indicating that MR CTLD4-8 is indeed related to the COS6-induced inflammation of macrophages.

### Mannone Receptor Bound COS6 Through CTLD4-8

**Figure 2:**
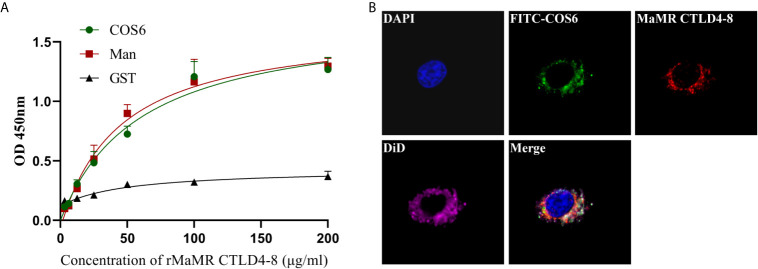
Interaction of chitooligosaccharide hexamer (COS6) to mannose receptor C-type lectin-like domain 4-8 of *Megalobrama amblycephala* (MaMR CTLD4-8). **(A)** ELISA analysis of the interaction between rMaMR CTLD4-8 and COS6. The microtiter plates were coated with carbohydrates (Man as a positive control) and incubated with the recombinant protein [glutathione-S-transferase (GST)-tag protein as a negative control]. After incubation with anti-MaMR CTLD4-8 antibody, the interaction was detected with goat anti-mouse immunoglobulin G (IgG)–horseradish peroxidase (HRP) conjugate at 450 nm. **(B)** Co-localization of COS6 and MaMR CTLD4-8 by indirect immunofluorescent assay. Primary macrophages were incubated with fluorescein isothiocyanate (FITC)-labeled COS6 for 30 min, 4% (v/v) paraformaldehyde-fixed, anti-MaMR CTLD4-8 incubated, labeled with Cy3, 4′,6-diamidino-2-phenylindole (DAPI), and 1,1-dioctadecyl-3,3,3,3-tetramethylindodicarbocyanine (DiD) staining, and the results were presented *via* confocal microscopy. Data were shown as means ± SD (n = 3). Scale bar:10 μm.

To illustrate COS6 as a recognized ligand of MR, the molecular level assay was performed by ELISA, and the cell level assay was demonstrated by indirect immunofluorescence. In the molecular level assay, OD450 values reflecting the binding ability of rMaMR CTLD4-8 with COS6 or D-mannose increased with the elevation of the concentration of rMaMR4-8, indicating that MaMR CTLD4-8 can bind to COS6 and D-mannose in a dose-dependent manner. GST-tag protein was used as a negative control, and its OD450 was maintained at about 0.3 without significant change, which indicates that GST-tag protein could not bind COS6 or D-mannose (**Figure 2A**). In the cell level assay, immunofluorescence microscopy was performed to examine the locations of MaMR CTLD4-8 and COS6 in macrophages. The results indicate that COS6 and MaMR CTLD4-8 co-localized in macrophages (**Figure 2B**).

### COS6 Was Internalized Into Macrophages

**Figure 3:**
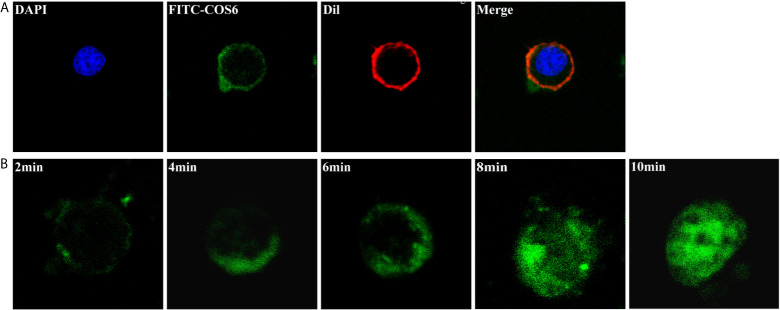
Internalization of chitooligosaccharide hexamer (COS6) in macrophages. **(A)** Here, 0.05 mM fluorescein isothiocyanate (FITC)-COS6 is internalized into macrophages by incubating for 10 min at 4°C, then the cell membrane was stained with 1,1’-dioctadecyl-3,3,3’,3’-tetramethylindocarbocyanine perchlorate (DiI) and the cell nucleus was stained with 4′,6-diamidino-2-phenylindole (DAPI). **(B)** The internalization of FITC-COS6 at different times (2, 4, 6, 8, and 10 min). Cells (1 × 10^6^–1 × 10^7^) were incubated with 0.05 mM FITC-COS6 at 28°C and examined by a confocal microscope. All the experiments were repeated at least three times. Scale bar:5 μm.

The kinetics of FITC-COS6 internalization in macrophages was visualized by confocal microscopy. The internalization of FITC-COS6 in blunt snout bream macrophages was temperature-dependent. Internalization at 4°C (**Figure 3A**) was a relatively slow uptake process compared to that at 28°C (**Figure 3B**). The internalization of FITC-COS6 by blunt snout bream macrophages was time-dependent. FITC-COS6 bound to the cell membrane first, then gradually internalized into the cell, and finally spread to the cytoplasm, and the fluorescence intensity also increased with time (**Figure 2B**).

### The Internalization of COS6 by Macrophages Was Mediated by Mannose Receptor CTLD4-8

**Figure 4:**
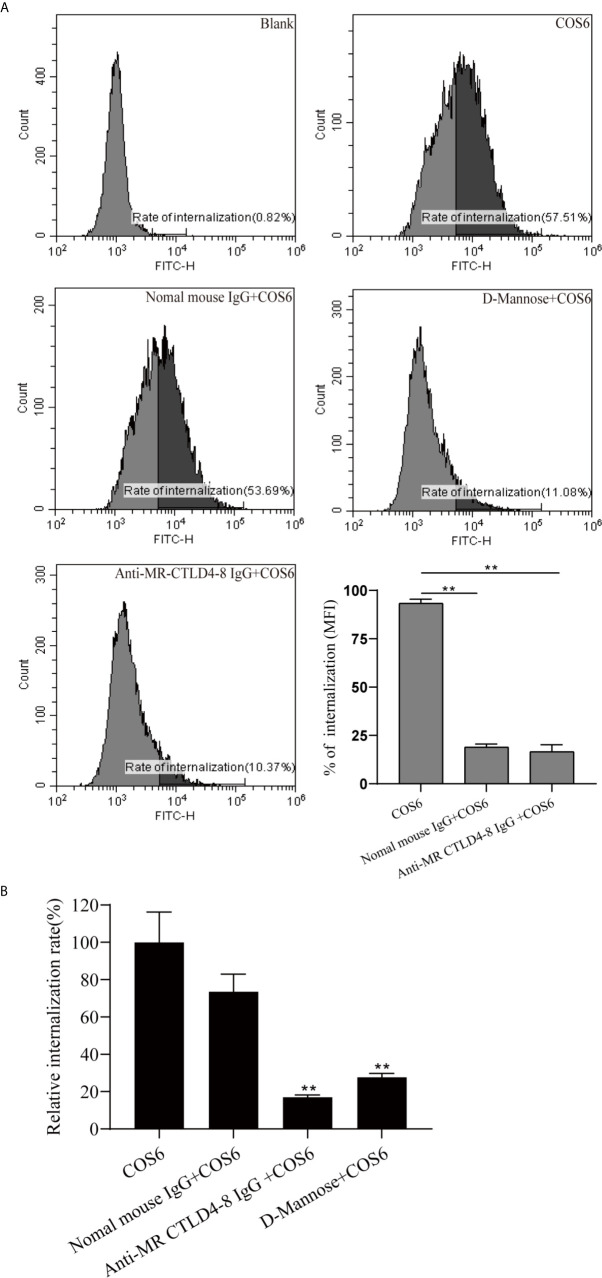
Internalization of fluorescein isothiocyanate (FITC)-chitooligosaccharide hexamer (COS6) by macrophages with or without blocking mannose receptor (MR) CTLD4-8. Anti-MR CTLD4-8 (4 μg/ml) and D-mannose (2 mg/ml) blockade of the cells for 2 h, and the macrophages were incubated with FITC-COS in a six-well plate at 28°C for 30 min. **(A)** The internalization rate of FITC-COS6 by flow cytometry. The mean fluorescence intensity (MFI) of internalization in macrophages was recorded. **(B)** Inhibitors of MR binding and function block the association of COS with primary macrophage. Cells were preincubated with 2 mg/ml D-mannose, mannose receptor C-type lectin-like domain 4-8 of *Megalobrama amblycephala* (MaMR-CTLD4-8) specific antibody, negative IgG, or medium alone. Then, 0.05 mM FITC-COS6 was added, and cells were incubated, washed, lysed, and mixed uniformly. Fluorescence intensity was measured. Data were shown as means ± SD and collected from three independent experiments, with (*) p < 0.05 and (**) p < 0.01 *vs.* the COS6 group (set as 100).

To determine the effect of MR CTLD4-8 in internalizing COS6 in the macrophages of blunt snout bream, we used anti-MaMR CTLD4-8 antibody to block the cells. The final result was presented by flow cytometry and fluorescence microplate reader. The internalization results of FITC-COS6 showed that compared with the blank group, the internalization rate of FITC-COS6 by macrophages that were not blocked reached 57.51%, and the internalization rate of the negative control group also reached 53.69%. As a positive control group, D-mannose reduced the internalization rate of FITC-COS6 to 11.08%, but the antibody pretreatment group also significantly reduced the internalization of FITC-COS6 by macrophages to 10.37% (**Figure 4A**). The fluorescence intensity measured by the fluorescence microplate reader was consistent with the trend of flow cytometry results (**Figure 4B**). These results confirmed that MaMR CTLD4-8 mediates the internalization of COS6 in blunt snout bream macrophages.

### The Internalization of COS6 in Macrophages Depends on Clathrin- and Caveolin-Mediated Pathways

**Figure 5:**
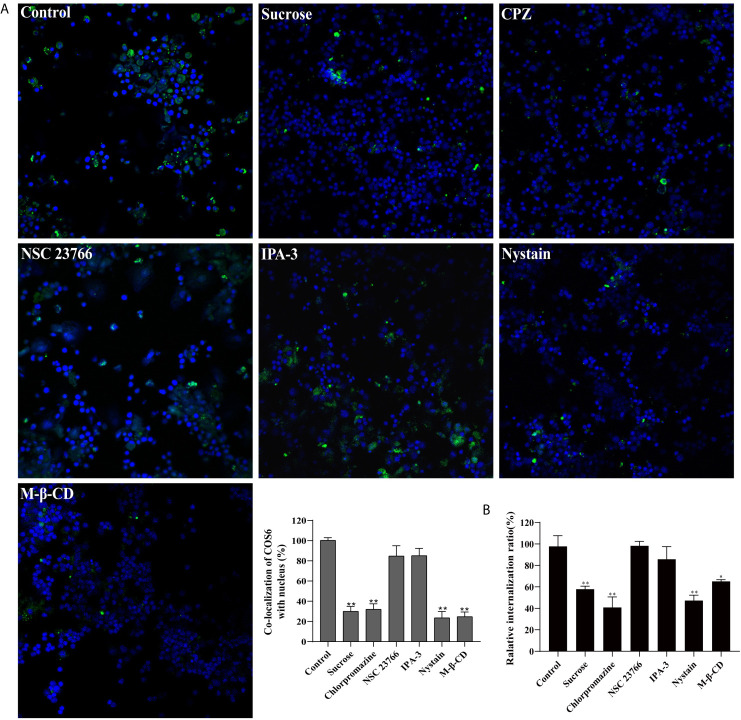
Effect of endocytic pathway inhibitors on chitooligosaccharide hexamer (COS6) uptake by macrophages. **(A)** Pretreat cells with endocytosis inhibitors or absence (control) and observe the uptake of fluorescein isothiocyanate (FITC)-COS6 by macrophages under a confocal microscope. The co-localization percentage of FITC-COS6 and nucleus was analyzed using WCIF ImageJ software. **(B)** Cells were treated with endocytosis inhibitors and incubated with FITC-labeled COS6, washed, lysed, and mixed uniformly. Fluorescence intensity was measured. Data were shown as means ± SD (n = 3), with (*) p < 0.05 and (**) p < 0.01 *vs.* the control group (set as 100). Scale bar: 250 μm.

To investigate the endocytosis of COS6 in the macrophages of blunt snout bream, we used endocytosis inhibitors to block clathrin-dependent (CPZ and sucrose), caveolin-dependent (M-β-CD and nystatin), and micropinocytosis (NSC23766 and IPA-3) pathways. Relative to the control group, confocal image showed that CPZ, sucrose, nystatin, and M-β-CD can effectively block the uptake of COS6 by macrophages, and NSC23766 and IPA-3 have no significant effect on the uptake of COS6 by macrophages (**Figure 5A**). By measuring the fluorescence intensity of ingested FITC-labeled COS6, the internalization rate relative to the control group was significantly reduced by clathrin and caveolin pathway inhibitors, reducing to 57.80% (sucrose), 40.90% (CPZ), and 47.27% (nystatin). Similarly, macropinocytosis pathway inhibitor had no significant effect (**Figure 5B**). It indicated that COS6 is internalized into macrophages through clathrin- and caveolin-dependent pathways.

### Toll-Like Receptor 4 and Mannose Receptor CTLD4-8 Coordinately Regulated the Pro-Inflammatory Response of Macrophages by COS6

**Figure 6:**
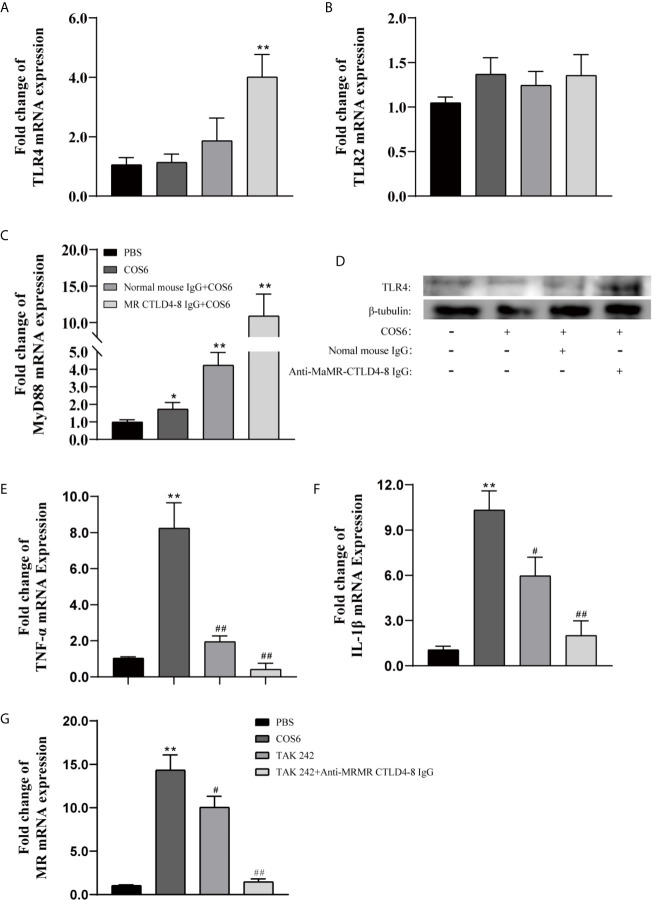
The expression of Toll-like receptor (TLR)2/4, mannose receptor (MR), and inflammation-related genes in chitooligosaccharide hexamer (COS6)-stimulated macrophages with or without blocking. **(A)** TLR4, **(B)** TLR2, **(C)** MyD88, **(D)** Western blot analysis of TLR4 and TLR2, **(E)** tumor necrosis factor (TNF)-α, **(F)** interleukin (IL)-1β, **(G)** MR. Western blot analysis using anti-TLR4, anti-TLR2 antibodies, and anti-β-tubulin antibody was used to evaluate the quantity of proteins in each lane. TLR2 has no significant difference; the data are not shown. Each experiment was executed in triplicate. Data were shown as means ± SD (n = 3), with (*) p < 0.05 and (**) p < 0.01 *vs.* the phosphate buffered saline (PBS) group, (^#^) p < 0.05 and (^##^) p < 0.01 *vs.* the COS6 group (set as 1).

Since the intracytoplasmic domain of MR is very short and does not contain signal transduction motifs (25) and previous results also indicated that MR CTLD4-8 is involved in the immune regulation of COS6-stimulated macrophages (**Figure 1**), other receptors are needed to explore the signal transduction involved in MR. Therefore, we investigated whether TLR2 or TLR4 were also involved in COS6-activated signal transduction pathway in blunt snout bream macrophages. In our results, compared with the PBS group, the gene expression of TLR4 was significantly upregulated after the MaMR CTLD4-8 antibody blocked the recognition of COS6 (**Figure 6A**), while the gene expression of TLR2 was not significantly changed (**Figure 6B**). The expression of MyD88, a downstream linker molecule of TLRs, was also significantly upregulated with TLR4 (**Figure 6C**). Normal mouse IgG was used as a negative control in the experiment. Similarly, at the level of protein expression, the expression of TLR4 was significantly upregulated after antibody blocking, while TLR2 did not change significantly (**Figure 6D**). To further investigate the signal transduction through TLR4-mediated signal pathway after COS6 activation in blunt snout bream macrophages, we used the TLR4 inhibitor TAK242 to block the intracellular TIR domain of TLR4 and block signal transmission. As shown in the figure, TAK242 significantly inhibited the inflammatory activation effect of COS6 on macrophages, and under the combined action of anti-MaMR CTLD4-8 and TAK242, the expression of TNF-α (**Figure 6E**) and IL-1β (**Figure 6F**) was significantly inhibited, and the expression level was reduced to the same level as that of the PBS group. Moreover, the expression of MR was also significantly inhibited by TAK242, and the expression level of MR was consistent with that of resting macrophages after both extracellular and intracellular signal recognition and transduction pathways were blocked (**Figure 6G**). These results indicated that MR and TLR4 were inseparable in COS6 activation of macrophages. Collectively, MR-mediated COS6-induced macrophage inflammation is regulated by the intracellular signal transduction pathway of TLR4. Previous studies have shown that COS6 activates macrophages through the MAPK/NF-κB pathway (24), suggesting that COS6 activates macrophages through the TLR4–MyD88–NF-κB signaling pathway, and is co-mediated by MR CTLD4-8 and TLR4.

## Discussion

Chitosan has become a popular adjuvant widely studied in aquaculture in recent years due to its nontoxic, biodegradable, good viscosity, and antimicrobial biological properties (26). In previous studies, we have confirmed that chitosan combined with IFN-γ can effectively activate grass carp (*Ctenopharyngodon idella*) macrophages (27) and is an aquatic adjuvant with excellent immune protection (28, 29). COS is an oligomer of chitosan. Compared with chitosan, it also has the characteristics of good water solubility and easy absorption. In mammals, COS is widely known to activate resting macrophages and has good immune effects in both pro-inflammatory and anti-inflammatory aspects (10, 15, 30). In previous studies, we have a certain understanding of the effect of COS on the immune activity of fish macrophages (24, 31), but the mechanism of COS on fish macrophages is still blank. As an important immune effector cell, macrophages can not only eliminate pathogens and presenting antigens but also release a large number of inflammation-related genes to participate in host defense and maintain tissue homeostasis (32). In recent years, the immunostimulating activity of COS *in vitro* has also been focused on macrophages. In this study, we used the head kidney macrophages of blunt snout bream as the experimental subject to demonstrate the important role of MR in COS6 activation of macrophages. We explored the recognition and binding ability of COS6 and MaMR CTLD4-8 at the molecular and cellular levels respectively and explored the internalization properties of COS6 in macrophages. Last but not least, the signal transduction pathway that COS6 activates macrophages has also been explained.

The most notable feature of MR is that a single peptide chain contains multiple CTLD domains. Among them, the CTLD4 domain can recognize specific carbohydrate components (mannose, fucose, N-acetylglucosamine, and other specific sugar residues), thereby binding to the carbohydrates containing these sugar residues mediates cellular immune regulation, for example, sugars on the surface of some pathogenic microorganisms (33, 34), allergens (35, 36), or polysaccharides of traditional Chinese medicine (TCM) (37, 38). However, CTLD4 requires two Ca^2+^ to be involved in the binding sugar ligand, and Ca^2+^ is related to the release of ligands from CTLD4 in the endosome. Although CTLD4 can bind monosaccharides alone, the binding of the entire receptor and ligand requires the participation of CTLD4-8 (39). N-acetylglucosamine is the smallest unit of COS, and CTLD4 of MR can specifically recognize and bind to the glucose residues of N-acetylglucosamine. However, N-acetylglucosamine has been shown to be unable to activate blunt snout bream macrophages in our previous studies (24). Our results showed that COS6 not only could activate the macrophages but also was specifically recognized and bound by MR CTLD4-8. The binding curve showed that the binding activity of COS6 and MR CTLD4-8 is almost the same as mannose, which was previously considered the best ligand. The binding of Ca^2+^ between MR and ligand in mammals is essential, and we have previously reported that MaMR CTLD4-8-mediated phagocytosis of bacteria is also Ca^2+^-dependent (40). We think that MaMR CTLD4-8 also requires the participation of Ca^2+^ in the recognition and binding of COS6. In addition, as same as macrophages MR-targeted nanocarriers or vaccines in recent years (41), COS6 is a ligand that can specifically bind to MR and is an excellent immune enhancer. Nanocarriers or vaccines targeting macrophage MR with COS as the material are worthy of further development.

MR is a highly effective endocytic receptor, which mediates the macrophages to uptake a variety of pathogenic microorganisms or ligands. MR is targeted into cells expressing MR by interacting with specific ligands (42). There have been many reports on MR-mediated internalization of soluble antigens, and they are often complexes or nanoparticles that have been glycosylated or modified by MR-specific ligands (43–45). MR recycles constantly between the plasma membrane and the early endosome (46). It relies on an aromatic amino acid motif in the cytoplasmic tail to guide the ligand-bound MR from the endosome to the plasma membrane (47). And MR-ligand is thought to circulate between the cell membranes through the clathrin pathway (48). In our results, it showed that COS6 could be internalized by macrophages of blunt snout bream in a time- and temperature-dependent manner. This is similar to the situation in mammals, except that accumulation of COS6 at the nucleolus has not yet been observed (20). And in the flow cytometry analysis, the internalization of FITC-labeled COS6 in macrophages was significantly inhibited after antibody blocking, proving that MaMR CTLD4-8 plays a key role in the internalization of COS6 by macrophages. Different from the mammalian MR-mediated endocytosis that is dependent only on clathrin, the MaMR CTLD4-8-mediated COS6 internalization is dependent both on clathrin and caveolin. The caveolin in the plasma membrane is often used as a marker protein molecule for lipid rafts (49). It not only participates in the transport of materials across the membrane but also contains a variety of key signal molecules that participate in cell signal transduction (50). This also suggests that the immune regulation of COS6 on macrophages is closely related to lipid rafts. After COS6 is internalized with MR CTLD4-8, it may be sent to the lysosome (51) or through the endosome to the Golgi apparatus (52), and the ligand is degraded after separation, to fully exert the immune regulatory effect of COS6.

Most immunostimulants bind specifically to receptors on the cell surface to activate cellular immunity. MR (12, 20), TLR4 (11, 53), and CR3 (15) are thought to mediate the immunostimulatory effect of COS on macrophages. In addition, TLR2 is also believed to be involved in the immune regulation of chitin (COS is its oligomeric derivative) on macrophages (54). Our results showed that the activation of COS6 on macrophages of blunt snout bream was closely related to MaMR CTLD4-8, which could mediate the expression of inflammation-related genes in macrophages. However, the expression of inflammation-related genes was not completely suppressed under the blocking of antibodies. It suggested that other receptors may also be involved in mediating the immune activation of COS6 on macrophages. The cytoplasmic tail of MR is extremely short and has no signal transduction motif (55). Therefore, it participates in signal transduction for immune regulation and requires the cooperation of other receptors. There have been many reports confirming that MR can coordinate with TLR2 or TLR4 to regulate the immune effect of cells in response to the stimulation of pathogenic microorganisms (25, 56, 57) or immune enhancers (38). Our results show that TLR4 is also involved in the MR-mediated immune response of macrophages to COS6. Combined with previous results (11), it is shown that the inflammatory response of COS-activated blunt snout bream macrophages involves MR and TLR4 and is regulated by the TLR4–MyD88–NF-κB signaling pathway. Although previously studied in human alveolar macrophages, *Pneumocystis* can promote the direct interaction of MR and TLR2, which leads to the release of IL-8 (56). However, we are still unclear about the interaction mechanism between the MR and TLR4. Lipid rafts serve as a platform for a variety of signal molecular interactions (58), and both pattern recognition receptors and lectin receptors in mammals are related to lipid rafts (59–61). Combining the previous results, lipid rafts may be the interaction and signal transduction platform for MR CTLD4-8 and TLR4.

In conclusion, this study explored the internalization mechanism and signal transduction pathway during the activation of blunt snout bream macrophages stimulated by COS6. The results showed that MaMR CTLD4-8 could specifically recognize and bind to COS6 and mediate COS6 into macrophages by both clathrin- and caveolin-dependent pathways, and MaMR coordinated with TLR4 to regulate the pro-inflammatory response of blunt snout bream macrophages. However, the interaction mechanism between MR and TLR4 remains elusive and needs to be further explored.

## Data Availability Statement

The original contributions presented in the study are included in the article/**Supplementary Material**. Further inquiries can be directed to the corresponding author.

## Ethics Statement

The animal study was reviewed and approved by Ethics Committee of Huazhong Agricultural University.

## Author Contributions

XL and AO conceived and designed the experiments. AO and HW performed the experiments and analyzed the data. AO, XL, and JS wrote the manuscript. All authors contributed to the article and approved the submitted version.

## Funding

This work was supported by the National Natural Science Foundation of China (31772879).

## Conflict of Interest

The authors declare that the research was conducted in the absence of any commercial or financial relationships that could be construed as a potential conflict of interest.

## Publisher’s Note

All claims expressed in this article are solely those of the authors and do not necessarily represent those of their affiliated organizations, or those of the publisher, the editors and the reviewers. Any product that may be evaluated in this article, or claim that may be made by its manufacturer, is not guaranteed or endorsed by the publisher.
