# Supplementary material for: Mannose Receptor Mediates the Activation of Chitooligosaccharides on Blunt Snout Bream (Megalobrama amblycephala) Macrophages
Source: Front Immunol. 2021 Aug 2;12:686846. doi: 10.3389/fimmu.2021.686846 (PMC8365301; doi:10.3389/fimmu.2021.686846)
Supplement: Supplementary file 1 [file DataSheet_1.docx]

Supplementary Material


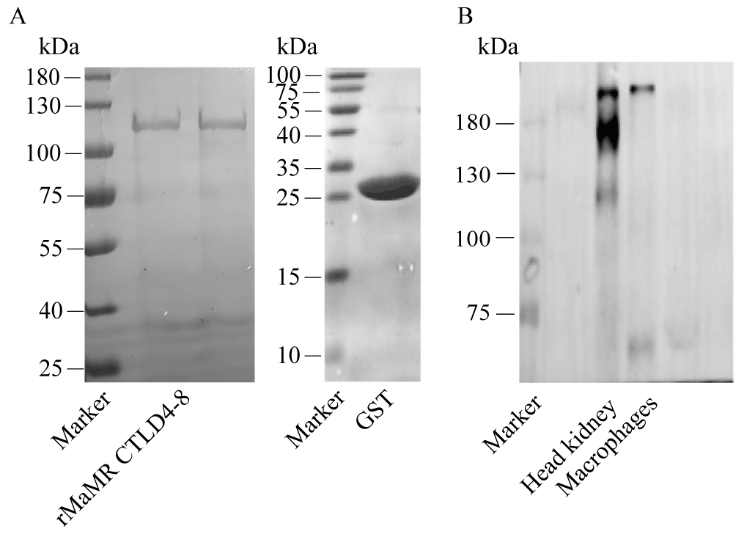


**Supplementary Figure 1.** Purification and antibody preparation of rMaMR CTLD4-8. (A) Purification of the MaMR CTLD4-8 recombinant protein and the GST tagged protein was analysed by SDS-PAGE. (B) The expression of anti-MaMR CTLD4-8 antibody in head kidney tissues and macrophages of *Megalobrama amblycephala* was analysed by western blot.


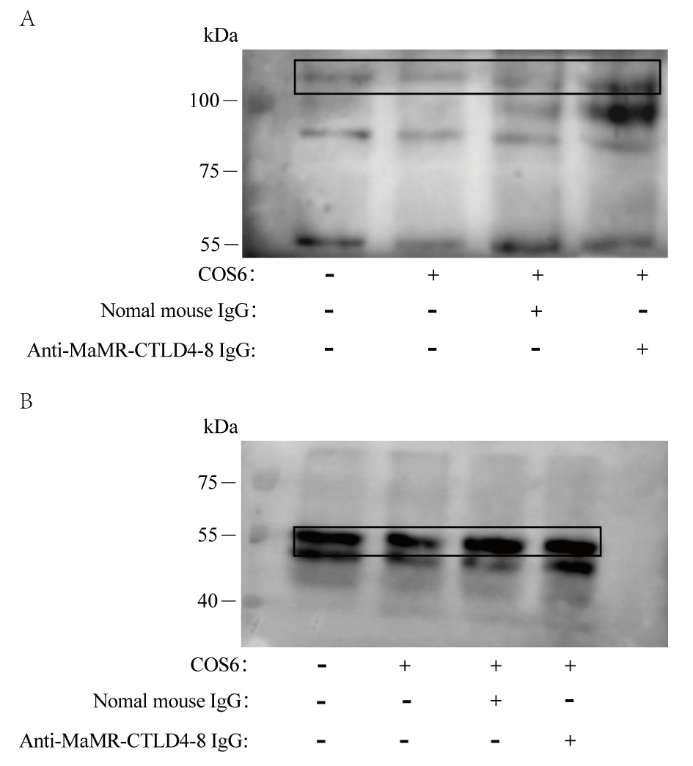


**Supplementary Figure 2.** The expression of COS6 on macrophages TLR4 (These are the original image of Figure 6D). Western blot analysis using anti-TLR4 (A), anti-TLR2 antibodies and anti-β-tubulin (B) antibody was used to evaluate the quantity of proteins in each lane. TLR2 has no significant difference, the data is not shown.
